# Diseases of the Army

**Published:** 1863-02

**Authors:** Sanford B. Hunt

**Affiliations:** Surgeon U.S.V.


ART.V.—*Diseases of the Army.* *By* Sanford B. Hunt, *Surgeon U. S. V.*

Before my second departure from Buffalo for the field, I inteneded a series of articles for your pages on the diseases of the army, as I had then met them. In sundry important matters of pathology and treatment, the opinions I then entertained have become convictions.

Especially is this so as to the lavish administration of quinine in fevers. I left New York with a regiment of 1000 men. At Baltimore one of the men came down with genuine Northern typhoid. He was placed in Camden Street Hospital, and there fed quinined and stimulated, through a serious illness to a satisfactory and rapid recovery. During his illness two or three of his family in the healthiest part of Tioga County died of typhoid; of course my man brought the fever from home with him.

Now that case is the only one of typhoid we have had in the regiment; yet we have treated something over a hundred cases of what is called typhoid here, successfully, too. About 120 cases of fever occurred. In four cases measles and consequent congestion of the lungs followed, or became intercurrent. All these died. In a fifth, diphtheria carried off the man during convalescence. This fever, then, if not typhoid, while generally called so, deserves some study; especially as it does not differ, in my opinion, from the fevers I observed upon the Peninsula in July and August last, and were there so fatal.

I am positive that the fever so prevalent in our camp is not typhoid, and, except upon the Peninsula, not remarkably malarial. Its negative signs are quite as important as its positive symptoms in making out the differential diagnosis. For a few days the patient feels weary and stupid while on duty; then has some pain in the back and headache; loses appetite, flushes up with fever, and about this time makes his appearance in hospital. He has a hot, dry skin; a pulse of about 100, frequently running higher, and a dry, brownish tongue. Otherwise he is pretty comfortable. Suffering little pain, he frequently maintains throughout a cheerful temper, and seems rather lazy than sick. As the disease progresses, diarrhœa commonly sets in, the pulse rarely exceeds 120, the teeth become covered with sordes, and the tongue is dry and cracked. Now comes a period of subsidence. The pulse declines in frequency and gains in fullness, the skin grows cool, appetite returns, and last of all the tongue regains its natural condition. A good appetite on a dry tongue was not uncommon. Recovery was complete and after that the man was unusually hardy, and, as we called it, *acclimated.* Such was the history of unmolested cases, in which Nature had her own way.

This fever was not typhoid, because 1st: There was diarrhœa, which was always beneficial to the patient, but never ulceration of Peyer's glands, never tympanitis, and never *exhausting* discharges. 2d: There was never coma or delirium. 3d: There were no rose-colored spots or sudamina. 4th: The treatment adapted to typhoid was positively injurious in this fever.

The treatment consisted of a cathartic, usually blue mass, on entering hospital. After that, the recumbent position and febrifuge drinks. The acetate of potassa was the favorite remedy of this class, given in doses of five grs. three or four times daily. When the tendency was a little toward depression, carbonate of ammonia was substituted. On this very simple and even insignificant treatment we relied with the utmost confidence. The duration of the disease was usually two or three weeks, sometimes longer. Quinine or cinchonine was only employed after the entire subsidence of the febrile action; then, in one grain doses before meals, it acted very happily, but during the fever, quinine uniformly increased the pulse and the general excitement. One of my assistants watched this phenomenon early in the epidemic with great care and precision. His experiments in a few cases settled the rule, and in more than a hundred fevers that followed, the quinine was only used as an adjuvant in convalescence. Alcoholic stimulants were always injurious.

I am fearful that the distinction I have been endeavoring to enforce between true typhoid, with its regular tendency towards death and its consequent demand for early supporting treatment, and the easy, simple, acclimating fever of the camp, has been badly neglected by many army surgeons. In the former, quinine and brandy are in the best sense of the word remedies; while in the latter they are literally poisons. Yet both are continued fevers, both have diarrhœa, sordes and dry tongue—*ergo*, both are commonly treated alike; while in fact, aside from these few resemblances the two fevers are as widely separated as it is possible to be, in character, pathology, tendency and treatment.

